# Characteristics of published mini and rapid health technology assessments reports: a cross-sectional analysis

**DOI:** 10.1017/S0266462326103493

**Published:** 2026-02-06

**Authors:** Sharon McLaughlin, Aanisa Abeer, Melissa K. Sharp, Kieran A. Walsh, Cassandra Nemzoff, Sheilagh Foley, Ed Clifton, Michelle Flood, Susan Spillane, Patricia Harrington, Conor Teljeur, Michelle O’Neill, Susan M. Smith, Máirín Ryan, Barbara Clyne

**Affiliations:** 1 Department of Public Health & Epidemiology, School of Population Health, Royal College of Surgeons in Ireland, Ireland; 2 Health Information and Quality Authority, Ireland; 3 School of Pharmacy, University College Cork, Ireland; 4 Department of Global Health and Development, London School of Hygiene & Tropical Medicine, UK; 5 Public and Patient Representative, Ireland; 6 Scottish Health Technologies Group, UK; 7 School of Pharmacy and Biomolecular Sciences, Royal College of Surgeons in Ireland, Ireland; 8 PPI Ignite Network, University of Galway, Ireland; 9 Discipline of Public Health and Primary Care, School of Medicine, Trinity College Dublin, Ireland; 10 Department of Pharmacology and Therapeutics, Trinity College Dublin, Ireland

**Keywords:** health economics, economic evaluation, health technology assessment, rapid, mini, HTA domains

## Abstract

**Objectives:**

While rapid health technology assessments (HTA) are important for decision makers, there is no universally accepted definition or standardized methods. The objective of this study was to analyze the content and conduct of published rapid and mini-HTA reports.

**Methods:**

We conducted a cross-sectional analysis of rapid and mini-HTA reports identified from the International HTA Database (2014–April 2024) and supplementary searches of HTA agency websites. We extracted descriptive data on rapid HTA products; specifically, which traditional HTA domains were included or omitted, evidence synthesis methods, and approaches to interest-holder (formerly known as stakeholder) engagement. Data analysis was conducted in Stata.

**Results:**

We included 203 rapid HTA reports. Most frequently included HTA domains were clinical effectiveness (99 percent) and safety (82.3 percent). Legal aspects (12.3 percent) and budget impact analyses (10.8 percent) were less frequently reported. Across reports reviewing clinical effectiveness (n = 201), generic literature searches were the most predominantly self-reported evidence synthesis method (37.8 percent), with updates (1.5 percent) and overviews (2 percent) of systematic reviews less common. Cost-utility analyses were the most commonly self-reported form of economic evaluation (36.2 percent). Additionally, public consultations (68 percent) were the most commonly reported ways to engage with interest holders.

**Conclusion:**

Our analysis highlights variations across rapid HTA reports and will contribute to wider research aiming to establish a clearer definition and framework of rapid HTAs and inform when and how rapid HTAs are performed. Clearer reporting and justification of simplifications in rapid HTA reports are needed.

## Key points

- To our knowledge, this study is one of the first to review the content of rapid HTA reports. Many reports prioritize clinical effectiveness, safety, and a description of the technology domains, while domains such as ethical, legal, and patient and social aspects were often simplified or omitted.
- While interest-holder engagement is often seen as a challenge in rapid HTA, at least three-quarters of the reports in our data set included some form of engagement, with public consultations and advisory groups most common.
- The replicability of rapid HTA reports may be improved by more consistent reporting of evidence synthesis and costing methods, as well as greater transparency in reporting of omissions and simplifications in approaches. Standardized guidelines are needed.

## Introduction

Health technology assessment (HTA) can be broadly defined as a multidisciplinary process that uses explicit methods to determine the value of a health technology (e.g., drugs, medical devices, surgical procedures, etc.) at different points in its life cycle (1). The purpose of HTA is to inform decision-making to promote an equitable, efficient, and high-quality health system (1). HTAs are produced in response to questions identified by decision-makers such as government healthcare ministries, regional health authorities, or hospital systems. These questions typically concern the clinical and cost-effectiveness of the technology and its impact on healthcare delivery (2). HTAs are also developed in response to manufacturers’ submissions, particularly for new drugs seeking reimbursement. The final HTA product varies in content, length, and methodological robustness, as HTA approaches and definitions are not globally standardized and accepted (1;3). However, the International Network of Agencies for Health Technology Assessment (INAHTA) defines a full HTA as always including the following domains and methodologies: the description of the technology and its current use; evaluation of safety and effectiveness; determination of cost-effectiveness, for example, through economic modeling (when appropriate); providing information on costs or budget impact; and discussion of organizational considerations (4). Producing full HTAs requires multidisciplinary expertise and is a lengthy process – an average of 9 months for a full HTA based on a 2013 survey (4); however, the process can extend over several years (5), and is dependent on many factors such as topic complexity, resource availability, and interest-holder (formerly known as stakeholder (6)) engagement.

The demand for HTAs by healthcare decision-makers has increased, accompanied by an increasing demand for faster responses to support timely decisions (7;8). In response to this demand, products such as rapid HTAs and mini-HTAs have been developed. There is a lack of standardization regarding the names of these products (4;7), with terms such as mini-HTA, rapid HTA, and rapid review often used interchangeably (see Table 1 for definitions). More recently, adaptive HTA (aHTA) has been used to describe approaches that adapt the standard HTA process to fit specific constraints, such as limited time, data, or capacity. These approaches (hereinafter referred to as rapid HTA) employ omissions or simplifications of full HTA. Although many HTA agencies produce rapid assessments in some form, there is considerable variation in both the terminology and the approaches used. The completion time for these rapid products is much faster on average (e.g., 4 months for a mini-HTA – based on 2013 data) (4); however, this again varies depending on factors such as the type of technology assessed, the decision makers’ questions, and the available evidence. These approaches can be highly responsive to the development of new technologies, potentially provide faster responses than full assessments, and allow for more efficient resource prioritization (9;10). At the same time, decision-makers demand reliable evidence to support timely decisions and expect the validity, reliability, and quality of rapid methods to come close to that of traditional “full” methods (11). Consensus regarding the methodological approaches and guidance for conducting rapid HTAs has been limited to date (12;13), resulting in significant variation across agencies in terms of what “rapid” means. For some forms of abbreviated HTAs, the reporting has been found to be inconsistent, poor quality, and lacking sufficient detail (14;15). Additionally, rapid methods are appropriate for certain technologies more than others, particularly in cases where there is less uncertainty regarding the evidence for the technology, or there is an urgent need for a decision (7). In these instances, full HTA approaches may not be necessary or possible.

**Table 1.** General characteristics of included reports (n = 203)

Variable	N (%)
Number of HTA agencies	21 (100)
Country/region	
Austria	2 (1)
Belgium	8 (3.9)
Canada	33 (16.3)
Croatia	1 (0.5)
Europe	24 (11.8)
Ireland	5 (2.5)
Malaysia	45 (22.2)
Norway	6 (3)
Scotland	1 (0.5)
Spain	29 (14.3)
Switzerland	3 (1.5)
Tunisia	1 (0.5)
United States	2 (1)
Wales	43 (21.2)
Product tag	
Mini-HTA^a^	78 (38.4)
Rapid review^a^	123 (60.6)
Rapid-HTA^b^	2 (1)
Types of technology	
Device (e.g. monitoring devices)	53 (26.1)
Digital health technology (e.g. electronic systems)	1 (0.5)
Drug	28 (13.8)
Multiple technologies	5 (2.5)
Procedures/therapeutic techniques (e.g. surgery)	67 (33)
Public health interventions (e.g. population level programs)	5 (2.5)
Screening programs (e.g. cancer screening)	6 (3)
Specialized clinical care (e.g. specialty care)	1 (0.5)
Telemonitoring (e.g. remote monitoring)	5 (2.5)
Test/diagnostic tools (e.g. rapid antigen tests)	28 (13.8)
Vaccine (e.g. vaccination programs)	4 (2)

a Product tags “mini-HTA” and “rapid review” correspond to the International HTA Database classifications. As per INAHTA Product Type Classifications, Mini-HTA will *always:* describe the characteristics and current use of the technology; evaluate safety and effectiveness issues; provide information on costs/financial impact, conduct a comprehensive systematic literature review, and critically appraise the quality of the evidence base. It will optionally address organizational considerations. A Rapid Review will always describe the characteristics and current use of the technology, and evaluate safety and effectiveness issues. A rapid review will often conduct a review of only high level evidence or of recent evidence, and will optionally critically appraise the quality of the evidence base and/or provide information on costs/financial impact.

b The “rapid-HTA” tags refer to the supplementary search, in which two agencies referred to their reports as “rapid HTAs” via email contact.

Another key challenge for rapid HTA approaches is interest-holder involvement. Interest-holder (6) (i.e., patients, policymakers, etc.) involvement in HTA processes brings benefits such as including relevant and transparent outcomes, understanding uncertainty of results, and enabling implementation (16). Yet how to effectively involve interest holders in rapid HTA processes, where short deadlines allow little time for input, has been identified as a challenge for HTA agencies (17). Additionally, determining the appropriate type of engagement in HTA adds further difficulty, influenced by factors such as the complexity of the topic, the impact of the final decision, and time and resource constraints (18). For example, a recent description from Argentina highlighted that while a public consultation process for rapid HTAs led to meaningful improvements in the final HTAs, this was supported by a part-time public consultation manager and a full-time administrative assistant, (19) resources which may not be available to many agencies. Previous studies reviewing methodological handbooks or surveying HTA agencies found variation in both terminology and HTA practices (4;7). These studies highlighted inconsistencies across organizations (4) or limited methodological detail (7). In order to understand current practice in rapid HTA internationally, we conducted a cross-sectional analysis with the aim of comparing and contrasting different published rapid HTA reports within the International HTA Database (produced by INAHTA) or published on HTA agency websites.

Our analysis is part of a wider research project to help establish when and how rapid HTAs should be performed. By examining published rapid HTA reports, we were able to observe how agencies are producing and reporting rapid HTAs in practice. This study is one of the first to provide insights into the variability of rapid HTA reports regarding included or omitted HTA domains, interest-holder engagement, and evidence synthesis methods. This analysis of international approaches may contribute to a clearer definition, framework, and role for rapid HTA.

## Methods

### Study design and data sources

This was a cross-sectional analysis of published rapid HTAs in the International HTA Database and is reported in adherence to the STROBE statement (20) (Supplementary Table 11). An a priori protocol was registered on Open Science Framework (21).

The International HTA Database entries contain information directly added by HTA agencies. We reviewed any reports tagged as “mini-HTA” or “rapid review,” and we also carried out a supplementary search of HTA agencies and references listed in a previous review of methodological handbooks (7). Additionally, we consulted our project advisory committee (PAC), which has expertise in evidence synthesis, health economics, HTA methodology, sociology, and pharmacy, and consulted our project-specific patient and public involvement (PPI) panel. Agencies included in the supplementary search are listed in our protocol (21). Title searches for relevant rapid HTA reports were conducted on HTA agency websites, and agencies were contacted to identify additional rapid HTAs.

We based our data extraction on the HTA Core Model® domains (see Supplementary Figure 1), which include a description of the health problem and technology, clinical effectiveness and safety, costs and economic evaluation, ethical and legal considerations, and organizational and social aspects (22). The model provides guidance for HTA reports, for example, it states that the costs and economic evaluation domain may involve a review of existing information (cost-effectiveness review), a de novo economic evaluation, and/or assessing affordability through a budget impact analysis (BIA).

### Data inclusion

We included reports with the following characteristics:

- Reports on the database that were tagged as “mini” or “rapid,” with references to titles such as “Rapid Health Technology Assessment,” “mini-HTA,” and “rapid review.”
- “De novo” (i.e., new rapid HTAs conducted, rather than assessments of existing rapid HTAs) rapid HTAs only. This included products where the HTA agency had conducted the synthesis of data themselves (e.g., a de novo economic analysis, systematic review, etc.). This is in contrast to HTA reports produced following a submission of a dossier of evidence, for example, as submitted by a health technology developer to a HTA agency in application for reimbursement (10). We focused on de novo rapid HTAs, as this research is part of a larger project to inform the methodology for HTA agencies undertaking rapid HTAs as opposed to performing rapid appraisal.
- Reports assessing at least two of the HTA Core Model® domains (22), as described above (section “Study design and data sources”).
- HTAs where the results of domains were reported as separate research questions or distinct domains.
- Completed reports. Ongoing reports were only included if a final version of the ongoing project was available on the organization’s website, but the International HTA Database entry had not been updated.

**Figure 1.**
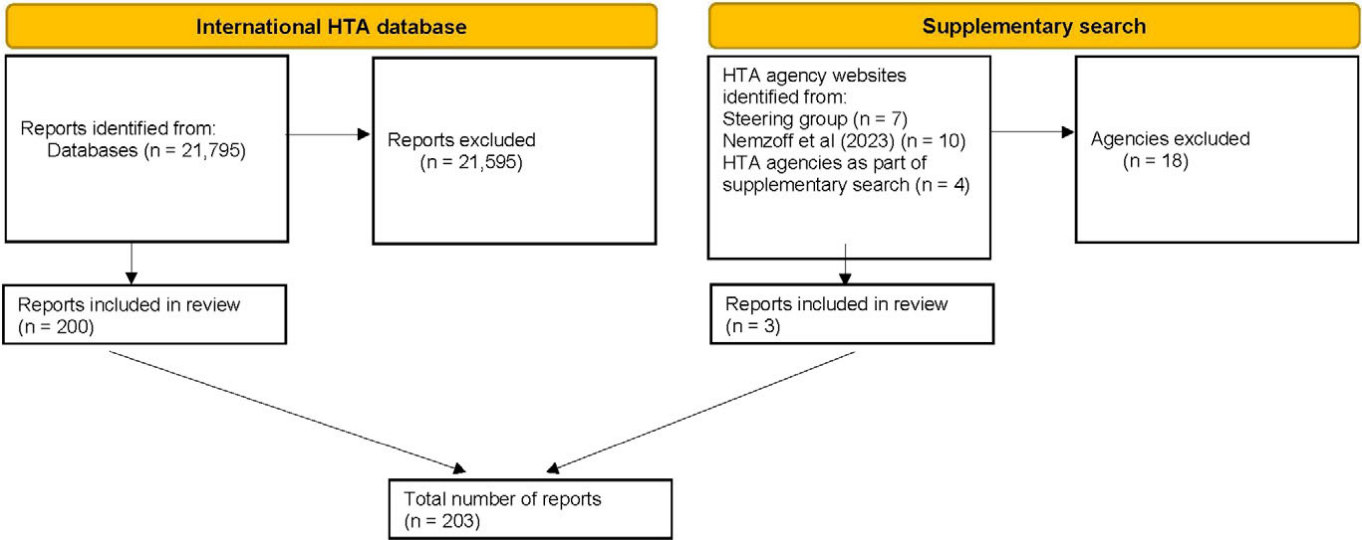
Flow diagram of included reports. HTA, health technology assessment. See Supplementary Table 1 for a full list of reasons for exclusions.

As rapid HTA is a relatively new concept (7), and in order to ensure relevance to current practice, only reports from 2014 up to the search date (April 2024) were considered for inclusion. Inclusion and exclusion are summarized in Figure 1.

### Data exclusion

We excluded any entry in the International HTA Database and supplementary search that did not meet the HTA definition as described in the introduction, including:

- Reports tagged as rapid or mini HTA but that were not assessing a health technology.
- Reports tagged as rapid or mini HTA focusing on only one of the HTA Core Model® domains (22), as listed above.
- Reports that were titled as stand-alone “systematic reviews.”
- Reports where an English language version could not be obtained. For non-English documents, we used DeepL (23) translation software and contacted relevant agencies to inquire about the availability of English translations. Where neither was possible, we excluded these documents.
- Reports that were not de novo but were rapid appraisals of HTAs.
- Reports that had not presented the results of domains as separate research questions or domains.

### Data extraction

Identified rapid HTA reports from the database were screened, selected, and extracted by two researchers (SML, AA). We extracted descriptive data on the types and scope of rapid HTA products; specifically, which full HTA domains were included or omitted (as per the HTA Core Model®), methods of evidence synthesis (e.g., systematic review or rapid review) (24), carried out within included domains, economic components, types of technology being evaluated, and approaches to interest-holder engagement. We extracted self-reported data on engagement and categorized it as either continuous engagement throughout the HTA (e.g., establishment of an expert advisory group or ongoing consultation of experts), one off engagements such as focus groups, interviews, or patient submissions, and finally, public consultations conducted at the end of the report drafting process. Domains were classified as full examinations, partial or unclear. Partial referred to instances where:

- Reports may have included HTA domains as an outcome in population, intervention, comparison, and outcome tables but did not treat these as separate domains or research questions.
- Domains were briefly discussed within other sections, without being treated as standalone topics.

We also recorded, where possible, the number of authors and coauthors and report characteristics such as page numbers, but due to inconsistencies in reporting, we have not presented these for analysis. All data were extracted as reported within each report. As it was outside of the research scope and there are currently no tools for assessing the overall quality of all HTA domains (e.g., no critical appraisal tool for organizational and social aspects), no quality appraisal was conducted.

All data were extracted to Microsoft Excel, with the data extraction form piloted and refined following feedback from our PAC.

### Data analysis and results

Data analysis was conducted using Stata software and descriptive statistics were used to report frequencies. Cross-tabulations were performed to examine the relationship between the type of technology versus included/omitted HTA domains and to examine the relationship between interest-holder engagement processes. Additionally, while not reported in the main text, an analysis of trends over time can be viewed in Supplementary Tables 2–7, while cross-tabulations between the report tag (i.e., mini and rapid) and the inclusion or omission of domains and interest-holder engagement are provided in Supplementary Tables 8 and 9. Rapid HTA reports were synthesized according to their agency, year, steps, purpose, as well as any general guidance on conduct and interest-holder involvement within selected reports.

## Results

A total of 21,795 reports were identified on the International HTA Database. After filtering results for “rapid” and “mini,” 21,359 results were removed. Of the 436 remaining results, 200 reports were deemed eligible for inclusion (Figure 1).

The supplementary search originally identified 17 additional HTA agencies that may have been performing rapid HTAs. Due to the lack of relevant results from title-based searches, each of these agencies was contacted once via email to enquire about relevant reports. Two agencies responded providing examples of a rapid HTA, while two agencies provided recommendations of four additional agencies to contact. In total, twenty-one HTA agencies were contacted, resulting in the identification of three eligible reports. Therefore, a total of 203 reports are included in this analysis.

### Report characteristics

A total of twenty-one HTA agencies across fourteen countries were included (Table 1). In total, 123 reports were titled as “rapid reviews,” seventy-eight reports as “mini HTAs,” and two were “rapid HTAs.” There were eleven different types of technology assessed, with procedures/therapeutic techniques being the most frequent (33 percent) (Table 1).

### HTA domains

Inclusion and omissions of HTA domains varied. The most commonly reported domains across the 203 reports were clinical effectiveness (99 percent), safety (82.3 percent), and the description and technical characteristics of the technology (78.8 percent). In contrast, economic evaluations (23.2 percent), ethical analyses (16.8 percent), legal aspects (12.3 percent), and the BIA component of the costs and economic evaluation domain (10.8 percent) were less common. Partial examinations of domains were most common for the health problem and current use of technology (48.8 percent) and the description and technical characteristics of the technology (21.2 percent). The inclusion of HTA domains was broadly similar across report tags, with clinical effectiveness being the most commonly included domain – appearing in 97.4 percent of reports tagged as mini-HTAs and 100 percent of reports tagged as rapid reviews (Supplementary Table 8).

Across health technologies, clinical effectiveness was reported in 100 percent of nine different categories, and safety was also widely reported, appearing in 95.5 percent of procedures/therapeutic technique reports, 92.9 percent of drug reports, and 88.7 percent of device reports (Table 2). In contrast, economic evaluations and the BIA component of the costs and economic domain were less common. BIAs were not reported in six of the displayed types of health technologies, and economic evaluations were not frequently reported across all types. Reviews of cost-effectiveness were most frequently reported in reports on drugs (89.3 percent), devices (71.7 percent), and procedures (64.2 percent). However, reports of these technologies placed less emphasis on legal (10.7, 17, and 11.9 percent) and ethical analyses (10.7, 22.6, and 13.4 percent).

**Table 2.** Type of health technology and included or omitted HTA domains (n = 203^a^)

Type of technology assessed, N (%)	HTA domains										
	CUR	TEC	EFF	SAF	ECO: cost review	ECO: evaluation	ECO: BIA	ORG	SOC	ETH	LEG
Device (n = 53)	36 (67.9)	52 (98.1)	53 (100)	47 (88.7)	38 (71.7)	16 (30.2)	7 (13.2)	39 (73.6)	33 (62.3)	12 (22.6)	9 (17)
Digital health technologies (n = 1)	1 (100)	1 (100)	1 (100)	0 (0)	1 (100)	1 (100)	0 (0)	1 (100)	1 (100)	0 (0)	0 (0)
Drug (n = 28)	3 (10.7)	12 (42.9)	28 (100)	26 (92.9)	25 (89.3)	1 (3.6)	1 (3.6)	9 (32.1)	5 (17.9)	3 (10.7)	3 (10.7)
Multiple technologies (n = 5)	3 (60)	5 (100)	5 (100)	3 (60)	3 (60)	1 (20)	0 (0)	2 (40)	1 (20)	1 (20)	0 (0)
Procedures/therapeutic techniques (n = 67)	31 (49.3)	52 (77.6)	67 (100)	64 (95.5)	43 (64.2)	18 (26.9)	9 (13.4)	34 (50.8)	28 (41.8)	9 (13.4)	8 (11.9)
Public health interventions (n = 5)	1 (20)	1 (20)	5 (100.	4 (80)	1 (20)	0 (0)	0 (0)	2 (40)	1 (20)	0 (0)	0 (0)
Screening programs (n = 6)	4 (66.7)	5 (83.3)	6 (100.0)	5 (83.3)	3 (50)	2 (33.3)	0 (0)	3 (50)	0 (0)	2 (33.3)	1 (16.7)
Specialized clinical care (n = 1)	0 (0)	1 (100)	1 (100)	1 (100)	0 (0)	1 (100)	0 (0)	1 (100)	0 (0)	0 (0)	0 (0)
Telemonitoring (n = 5)	2 (40)	4 (80)	4 (80)	3 (60)	4 (80)	0 (0)	2 (40)	4 (80)	3 (60)	2 (40)	2 (40)
Tests/diagnostic tools (n = 28)	20 (71.4)	23 (82.1)	28 (100)	11 (39.3)	14 (50)	6 (21.4)	3 (10.7)	17 (60.7)	11 (39.3)	5 (17.9)	2 (7.1)
Vaccine (n = 4)	1 (25)	4 (100)	3 (75)	3 (75)	3 (75)	1 (25)	0 (0)	3 (75)	1 (25)	0 (0)	0 (0)

a The table does not display reports that had unclear or partial examinations of HTA domains.

Abbreviations: BIA, budget impact analysis; CUR, the health problem and current use of technology; ECO, costs and economic evaluation; EFF, clinical effectiveness; ETH, ethical analysis; LEG, legal aspect; ORG, organizational aspects; SAF, safety; SOC, patient and social aspects; TEC, description and technical characteristics of the technology.

### Evidence synthesis methods

Across the domains where evidence synthesis is most applicable, that is, clinical effectiveness, safety, and cost-effectiveness, evidence synthesis methods varied (Table 3). For example, systematic reviews were reported in 20.9 percent of reports assessing clinical effectiveness, 22.8 percent of safety, and 17.8 percent of cost-effectiveness reports, while rapid reviews were reported in 11 percent of reports on clinical effectiveness, 9.6 percent of safety, and 14.8 percent of cost-effectiveness. Overviews of systematic reviews and updates to existing systematic reviews were described less frequently, with overviews appearing in only 2 percent of clinical effectiveness reports and in no reports on safety or cost-effectiveness. Similarly, updates of systematic reviews were reported in just 1.5 percent of clinical effectiveness reports and 1.2 percent of safety reports, and in no reports for cost-effectiveness. Alternative methods such as multiple types of evidence synthesis were reported in 1 percent of reports in clinical effectiveness reports, 0.6 percent of safety reports, and no reports for cost-effectiveness.

**Table 3.** Types of evidence synthesis as described by agencies within reports

Evidence synthesis method, N (%)	Clinical effectiveness (n = 201^a^)	Safety(n = 167^a^)	Cost-effectiveness (n = 135^a^)
Generic literature search	76 (37.8)	66 (39.5)	47 (34.8)
Systematic review	42 (20.9)	38 (22.8)	24 (17.8)
Rapid review	22 (11)	16 (9.6)	20 (14.8)
Overview of systematic review	4 (2)	0 (0)	0 (0)
Update of existing systematic review	3 (1.5)	2 (1.2)	0 (0)
Alternative methods^b^	2 (1)	1 (0.6)	0 (0)
Other^c^	28 (13.9)	21 (12.6)	22 (16.3)
Not described^d^	24 (11.9)	23 (13.8)	22 (16.3)

a The table does not display reports that had unclear examinations of domains.

b “Alternative methods” refers to reports that conducted multiple types of evidence synthesis for domains; such as a scoping review and a systematic review.

c “Other” refers to reports that had discrepancies in how the reviews were described.

d “Not described” refers to reports that conducted some type of evidence synthesis, but did not specify the method used.

Other reports were less specific, describing generic literature searches where methods were described in broad terms such as “literature search/review,” “systematic search,” “systematic literature search,” or a “hand search.” Additionally, several reports did not specify the type of evidence synthesis methods used, and some included unclear or inconsistent descriptions. In total, generic literature searches were the most commonly reported type of evidence synthesis used, appearing in 37.8 percent of clinical effectiveness, 39.5 percent of safety, and 34.8 percent of cost-effectiveness.

### Economic components

Across all reports (n = 203), a cost-effectiveness review was the most commonly reported component (66.5 percent), followed by an economic evaluation (23.2 percent), and a BIA (10.8 percent). It was unclear in 7.9 percent of reports if an economic evaluation was conducted and unclear in 5.4 percent of BIA cases. A total of forty-seven reports described at least one form of economic evaluation. Commonly self-reported types (Table 4) were a cost-utility analysis (36.2 percent) and a cost-effectiveness analysis (27.7 percent). Less common evaluations included cost-minimization (4.3 percent), cost-consequence (4.3 percent), and cost–benefit analyses (2.1 percent). Several reports indicated conducting a general cost analysis (21.3 percent).

**Table 4.** Types of economic evaluations as described by agencies within reports (n = 47^a^)

Economic evaluation	N (%)
Cost-utility analysis	17 (36.2)
Cost-effective analysis	13 (27.7)
General cost analysis	10 (21.3)
Multiple analyses	2 (4.3)
Cost-minimization analysis	2 (4.3)
Cost-consequence analysis	2 (4.3)
Cost–benefit analysis	1 (2.1)

a The table does not display reports that had unclear examinations of economic evaluations.

Out of 203 reports, 128 did not report conducting any form of economic evaluation or BIA. We found that 90.6 percent of these reports did not detail a justification for this. Additionally, 3.9 percent cited the reasons as “beyond project scope,” while the remaining reports cited issues such as “lack of clinical evidence,” “lack of evidence,” “lack of health economic evidence,” “lack of local data,” “lack of robust data,” “no legal obligation,” and “technology has not been marketed, manufacturer unable to provide cost data” (all at 0.8 percent).

### Interest-holder engagement

A total of 191 reports detailed some form of interest-holder engagement, while twelve reports did not detail any type. Additionally, it was unclear in thirteen reports if an advisory group/ expert opinion was established, and twenty-three reports were unclear if a public consultation occurred. Public consultations (68 percent) and advisory groups/expert opinion (59.1 percent) were the most common way to engage with interest holders. Contact from manufacturers (19.2 percent), patient groups (i.e., patient organizations or charities) (4.9 percent), interviews (4.9 percent), and focus groups (1 percent) were less common. Manufacturer contact/submission and patient group submissions were more commonly described in reports with advisory groups/expert opinion. The most common form of interest-holder engagement differed across report tags, with public consultations most frequent among reports tagged as mini-HTAs (85.9 percent) and advisory group/ expert opinion most frequent among reports tagged as rapid reviews (78.9 percent), Supplementary Table 9. Additionally, public consultations were more commonly reported in reports without advisory groups (81.4 percent) compared to those with advisory groups (63.3 percent) (Supplementary Table 10).

## Discussion

### Summary of results

Our examination of 203 rapid HTA reports across twenty-one different HTA agencies demonstrates international trends in rapid HTA reporting. The majority of included rapid HTAs were conducted on procedures/therapeutic techniques and medical devices. Clear trends emerged in the inclusion of certain HTA domains: clinical effectiveness, safety, and the description and technical characteristics of the technology were consistently reported regardless of the type of technology, while BIAs, economic evaluations, and ethical and legal aspects were less frequently reported. Drugs, devices, and procedures reports tended to prioritize cost considerations, however, reports assessing devices displayed a higher reported emphasis on patient and social aspects. Several reports were unclear in their descriptions of evidence synthesis methods, costing methods, and interest-holder engagement processes. Many reports used general terms for evidence synthesis methods (i.e., literature reviews), while others were more specific in their descriptions (e.g., systematic reviews, rapid reviews). Public consultation and advisory groups/expert opinion were the most commonly reported ways to engage with interest holders, with patient group submissions and focus groups being the least common.

### Comparison with other studies

Regional differences related to time to publication for drug assessments, methods of evaluation, and inclusion of patient preference data have been identified in HTA and reimbursement processes previously (25;26;27;28). Rapid methods have often been cited as lacking standardization, with differing approaches and methods adopted (7;12;13). It is perhaps unsurprising that we identified variation in published rapid HTAs in terms of inclusion and omission of HTA domains, evidence synthesis methods, and interest-holder engagement. As previously discussed, we also found inconsistencies and ambiguity in descriptions of costing methods, evidence synthesis methods, and interest-holder engagement.

However, we did find similarities across health technology types with clinical effectiveness, the description and technical characteristics of the technology, and safety consistently reported, indicating some consensus internationally on the inclusion of these domains within rapid HTA. This may align with some existing frameworks such as the HTA Core Model®, but may also be reflective of approaches such as rapid relative effectiveness assessments, which identify these domains as the minimum set for inclusion. These assessments were developed to produce HTA information within limited timeframes (22). Joint clinical assessments have also emerged from this framework, though these focus on appraisal of dossiers of evidence submitted by health technology developers (29). Unsurprisingly, the description of technology domain may have been included almost universally to define what the payer is funding.

Within our data, the most commonly cited approach to evidence synthesis in the clinical effectiveness and safety domains was classified as generic literature searching, highlighting the need for clearer reporting of evidence synthesis methods in rapid HTA. While methods such as overviews of reviews and updates to systematic reviews have been proposed as efficient options in comparison to de novo systematic reviews in HTA (8;30), we did not observe much use of these methods in our data. This may reflect a number of issues, including a lack of guidance for overviews of reviews during the study time period (31), a lack of expertise in the application of these methods, or an absence of existing reviews for the HTA topic – particularly for new and emerging health technologies. The adoption of these methods may reduce duplication of effort and increase collaboration between HTA agencies. Although rapid reviews provide a quicker synthesis of evidence, their use in HTAs may be contentious. (32) While some agencies are adopting rapid reviews (8), a limited number of reports are specifically labeling their evidence synthesis as such. Guidance for rapid reviews has likewise been in development during the study time period. Recent updated recommendations from the Cochrane Rapid Reviews Methods Group (33) and a series of guidance papers on methodological approaches in rapid reviews (34;35;36;37) may help strengthen their application as part of an overarching rapid HTA approach.

Cost-effectiveness reviews were reported in over 60 percent of included reports while de novo economic evaluations were conducted in less than a quarter of reports. There are many reasons why an economic evaluation may not be required (e.g., not relevant, no data, pre-existing evidence, outside the agency’s remit). However, 90.6 percent of included reports did not provide a justification, highlighting the need for greater transparency on omissions and simplifications in rapid HTA approaches. Similar to the literature (38), domains such as patient and social aspects, ethical, and legal analyses were excluded, likely due to resource and time constraints (39), the complexity of assessing these domains (40) or a lack of requirement from the HTA requester. Even “full” HTAs, may only reference, rather than analyze ethical considerations (41). It is likely rapid HTAs also omit these sections due to a lack of guidance to support conducting such analyses in a rapid context or perhaps the judgement that these aspects are not particularly contested in the context of the technology under assessment.

As previously discussed, one of the biggest challenges facing HTA agencies is designing an effective process for engaging interest holders, primarily patients and clinicians (17). Patient and public involvement (PPI) in HTA has broadly been characterized as either having patients as members of decision-making groups or through public consultation (42). Our data reported a similar pattern with patient representatives involved in advisory groups or consulted via interviews and focus groups, but there was a low reported number of patient group submissions (4.9 percent). Although interest-holder engagement is recognized as a challenge in HTA (17), our analysis conveys that approximately three quarters of our dataset included some evidence of engaging with interest holders, highlighting the importance of this aspect within rapid HTA.

### Strengths and limitations

To our knowledge, this study is one of the first to provide insights into the variability of rapid HTA reports regarding included or omitted HTA domains, interest-holder engagement, and evidence synthesis methods, as reported in publicly available rapid HTA reports. Using the International HTA Database as a primary data source provided us with access to a broad range of current reports. However, the database is not a complete list of HTA agencies, uploading to the database is voluntary, and agencies self-select report tags (e.g., full HTA, rapid review, mini HTA). While we carried out a supplementary search of references and organizations cited by Nemzoff et al. (7) and our PAC network, it is possible that not all HTA agencies conducting rapid HTAs were included in the analysis. Those agencies that are indexed in the database may have approaches to rapid HTA that are not reflective of broader HTA agencies and activities. Additionally, agencies (or us as authors) may “misclassify” their report types due to limited tag options. We were also unable to translate three reports. Finally, in discussion with our PAC, we applied the criterion that a report must contain at least two of the HTA Core Model® domains to qualify for inclusion in an effort to exclude single-domain systematic reviews. Therefore, certain methods or approaches could potentially be underrepresented in our sample.

For data extraction, we relied directly on the descriptions of evidence synthesis methods, economic evaluations, and other components provided in the reports, without interpreting their methods or terminology. As terminology may vary across agencies, it is possible that some discrepancies may exist in the data. We did not contact agencies to verify report content or conduct any critical appraisal, given the lack of suitable tools. Our research lays the groundwork for further research to establish a standardized framework for rapid de novo HTAs, which may enable future research to focus on the overall quality of rapid HTAs. A data dictionary detailing pragmatic decisions is provided in the data extraction file (21).

### Implications

Our analysis has highlighted that clinical effectiveness, the description and technical characteristics of the technology, and safety are generally reported domains within rapid HTA, indicating some consensus internationally on their inclusion. This inclusion may reflect consensus on the importance of these domains but could also be influenced by data availability. The evidence synthesis methods reported in these domains tended to more generic approaches, rather than rapid reviews or overview methods – which may be expected simplifications in a rapid HTA process. Scope for application of rapid and overview methods within rapid HTA should be explored. Rather than simplified versions, we found that domains such as ethics and legal aspects were omitted most often. There are several possible reasons for this: these domains may not be necessary depending on the technology type, there may be a consensus that these domains are less essential when time is short, or there could be uncertainty about how to incorporate them efficiently. If the latter, further guidance on how these domains can be included in a rapid approach would be useful. The series of guidance papers on methodological approaches in rapid reviews from the Cochrane Rapid Reviews Methods Group (34;35;36;37) serve as a strong foundation for developing such guidance. We also found economic evaluations were often not conducted, but a justification as to why was often not provided. We have compiled a table of recommendations for reporting rapid HTAs, which is provided in Supplementary Table 12. Overall, detailed reporting and justification of the simplifications/omissions in rapid HTA reports should be improved to support transparency and replicability (43;44). We hope this article will spark discussion about the nature of rapid HTA, encouraging greater clarity around its methods and, in turn, helping to shape realistic expectations about what rapid HTA can and cannot achieve.

## Conclusions

This cross-sectional analysis provides an overview of how international agencies perform rapid HTAs, contributing significantly to their understanding. While it was not possible to account for every HTA agency that performs rapid HTAs in our analysis, our sample does convey that rapid HTA is widely used across countries, and that clinical effectiveness, the description and technical characteristics of the technology, and safety are generally reported domains. There is variability in the inclusion or omission of other HTA domains, types of evidence synthesis, costing methods, and interest-holder engagement processes. Overall, consistent reporting of evidence synthesis and costing methods, and detailed reporting and justification of the simplifications/omissions in rapid HTA reports should be improved to support transparency and replicability. This analysis lays the groundwork for our further research which aims to establish a standardized framework for rapid de novo HTAs.

## Supporting information

10.1017/S0266462326103493.sm001 McLaughlin et al. supplementary material McLaughlin et al. supplementary material

## Data Availability

Most of the data supporting the findings of this study are available within the paper and its supplementary materials. Additionally, the data extraction file is available on the Open Science Framework (21).

## Abbreviations

BIA: budget impact analysis
CUR: the health problem and current use of the technology
ECO: costs and economic evaluation
EFF: clinical effectiveness
ETH: ethical analysis
EUnetHTA: European Network for Health Technology Assessment
HEOR: Health Economics and Outcomes Research
HIQA: Health Information and Quality Authority
HTA: health technology assessment
INAHTA: The International Network of Agencies for Health Technology Assessment
JCA: Joint Clinical Assessment
LEG: legal aspects
ORG: organizational aspects
PAC: Project Advisory Committee
PICO: population, intervention, comparison, and outcome
PPI: patient and public involvement
RCSI: Royal College of Surgeons in Ireland, University of Medicine and Health Sciences
REA: rapid relative effectiveness assessments
HTA: health technology assessment
SAF: safety
SOC: patient and social aspects
TEC: description and technical characteristics of the technology

## Supplementary material

The supplementary material for this article can be found at http://doi.org/10.1017/S0266462326103493.
